# Association between Pre-pregnancy BMI and Pregnancy Outcomes in Women with Gestational Diabetes: A Retrospective Cohort Study

**DOI:** 10.12669/pjms.41.1.10920

**Published:** 2025-01

**Authors:** Lianghui Zheng, Qiuping Liao, Jiaoxia Liu, Jun Shi, Zihua Chen, Lin Lin

**Affiliations:** 1 Lianghui Zheng Department of Obstetrics and Gynecology, Fujian Maternity and Child Health Hospital, College of Clinical Medicine for Obstetrics & Gynecology and Pediatrics, Fujian Medical, University, Fuzhou, Fujian, China; 2 Qiuping Liao Department of Obstetrics and Gynecology, Fujian Maternity and Child Health Hospital, College of Clinical Medicine for Obstetrics & Gynecology and Pediatrics, Fujian Medical, University, Fuzhou, Fujian, China; 3 Jiaoxia Liu Fujian Medical University, Fuzhou, Fujian Province, China; 4 Jun Shi Department of Obstetrics and Gynecology, Fujian Maternity and Child Health Hospital, College of Clinical Medicine for Obstetrics & Gynecology and Pediatrics, Fujian Medical, University, Fuzhou, Fujian, China; 5 Zihua Chen Department of Obstetrics and Gynecology, Fujian Maternity and Child Health Hospital, College of Clinical Medicine for Obstetrics & Gynecology and Pediatrics, Fujian Medical, University, Fuzhou, Fujian, China; 6 Lin Lin Department of Obstetrics and Gynecology, Fujian Maternity and Child Health Hospital, College of Clinical Medicine for Obstetrics & Gynecology and Pediatrics, Fujian Medical, University, Fuzhou, Fujian, China

**Keywords:** Pre-pregnancy BMI, Gestational Diabetes Mellitus, Pregnancy Outcomes, Pregnancy-Induced Hypertension, Low Birth Weight, Macrosomia

## Abstract

**Background & Objective:**

The specific influence of the pre-pregnancy body mass index (PPBMI) on women with gestational diabetes mellitus (GDM) is unclear. Our objective was to investigate how PPBMI categories affect pregnancy and neonatal outcomes in women with GDM.

**Methods:**

A retrospective cohort study was conducted using data from patients attending the Fujian Maternity and Child Health Hospital (Fuzhou, China) from 2021 to 2023. The participant records were stratified into four groups according to their BMI values: underweight, normal-weight, overweight, and obese. The pregnancy and neonatal outcomes for these BMI categories were analyzed using multivariable logistic regression.

**Results:**

The study included data from 2,909 pregnant women diagnosed with GDM. Underweight women with GDM showed significantly lower risks of pregnancy-induced hypertension (PIH) (adjusted OR, 0.26) and cesarean sections (adjusted OR, 0.55) but higher risks of low body weight (LBW) infants (adjusted OR, 3.40). Overweight and obese women experienced higher risks of PIH (adjusted OR, 2.96), cesarean sections (adjusted OR, 1.62), and macrosomia (adjusted OR, 1.43).

**Conclusion:**

PPBMIs significantly impact pregnancy outcomes in women with GDM. Both underweight and overweight/obese categories are associated with adverse outcomes, highlighting the need for pre-pregnancy counseling and interventions to achieve and maintain a healthy BMI.

## INTRODUCTION

The number of obese individuals has increased globally, leading to numerous health challenges, including adverse pregnancy outcomes.^1^,^2^ The World Health Organization (WHO) has issued statistical data showing that more than 2.8 million people die annually due to obesity-related complications.^3^ While much research has focused on the effects of a high body mass index (BMI) on pregnancy outcomes, the implications of low BMIs have not been as extensively studied. This knowledge gap is particularly relevant in populations where cultural and socioeconomic factors may contribute to a higher prevalence of underweight women.

Gestational diabetes mellitus (GDM) is a common occurrence characterized by glucose intolerance manifesting during pregnancy.^4^ GDM poses significant risks to both mother and child, such as increased chances of preterm birth, cesarean delivery, macrosomia, and future metabolic disorders.^5^,^6^ Understanding the association between pre-pregnancy BMI and GDM is crucial to develop targeted interventions to improve maternal and neonatal health outcomes.^7^,^8^ China has experienced significant dietary habits and lifestyle changes, contributing to a dual burden of malnutrition, with both undernutrition and obesity prevalent among women of reproductive age.^9^

High pre-pregnancy BMIs have been associated with high risks of gestational diabetes, postpartum hemorrhage, cesarean section, and fetal macrosomia.^10^,^11^ However, these studies often overlook the adverse outcomes associated with low pre-pregnancy BMI values, such as preterm delivery and low birth weight. Additionally, many studies have disregarded the variability in BMI classifications and the specific health characteristics of different cultural and geographical contexts.^12^ Studies specifically examining the association between pre-pregnancy BMI and pregnancy outcomes in women with GDM are lacking. The resulting literature gap is significant, as women with GDM face unique health challenges and risks influence by their pre pregnancy BMI. Addressing this gap is crucial for developing effective interventions tailored to this high-risk group. Herein, we tried to assess the potential associations between pre-pregnancy BMI categories and pregnancy outcomes in women with GDM to understand their effects on maternal and neonatal outcomes.

## METHODS

Clinical records of 2,909 pregnant women diagnosed with GDM who had given birth between 2021 and 2023 were retrospectively selected from the Fujian Maternity and Child Health Hospital database. Informed consent was waived due to the retrospective nature of the study, and the use of anonymized historical medical records data. All personal information was de-identified before the analysis to ensure privacy and confidentiality.

### Ethical Approval:

The institutional Medical Ethics Committee approved the study (approval number, 2024KY200, Final Approval Date: 2024-Aug-14).

### Inclusion criteria:

- A diagnosis of GDM.
- Singleton pregnancy.
- Complete medical records.

### Exclusion Criteria:

- Women with twin or multiple pregnancies.
- Spontaneous miscarriage or stillbirth.
- Pre-existing diagnosis of hypertension or diabetes mellitus.
- Thyroid disorders, viral infections, tuberculosis.
- Previous cesarean-section.
- Incomplete BMI data.

### Data Collection:

Data from the hospital database included three types of variables:

- Maternal characteristics, such as age, gravidity, parity, preterm history, height, pre-pregnancy weight, pre-pregnancy BMI, weight at delivery, weight gain, BMI group at delivery (categorized as underweight, appropriate pregnancy, or overweight), and pre-pregnancy BMI grouping (categorized as low, overweight, obese, or normal);
- Pregnancy outcomes, such as cesarean section, preterm birth, pregnancy-induced hypertension (PIH), GDM, hypothyroidism or hyperthyroidism, placental abruption, and premature rupture of m embranes (PROM)*;*
- Neonatal characteristics and outcomes, including sex, low birth weight (LBW), macrosomia, gestational weeks at birth, large-for-gestational-age (LGA), small-for-gestational-age (SGA), pre-term birth, neonatal jaundice, NICU admission, asphyxia, and chorioamnionitis.
- We calculated BMIs (kg/m^2^) as the ratios of weight (kg) divided by height squared (m^2^). We formed groups of patients on the basis of their pre-pregnancy BMI (PPBMI) values as follows:^13^ Underweight (BMI < 18.5 kg/m^2^); Normal weight (BMI between 18.5 kg/m^2^ and 24.0 kg/m^2^); Overweight (BMI between 24.0 kg/m^2^ and 28.0 kg/m^2^); Obese (BMI ≥ 28.0 kg/m^2^). We used data from the group of women with normal BMI (18.5–24.0 kg/m^2^) as the reference.

### Statistical Analysis:

We used SPSS Statistics for Windows, version 25.0 (IBM Corp, NY, USA) for statistical analyses. A cumulative logistic regression model was applied to identify the factors affecting PPBMI and gestational weight gain while controlling for confounding factors. Multivariate logistic regression models, which included cumulative and multinomial logistic regression, were used to adjust for confounders and determine independent risk factors for adverse maternal and neonatal outcomes. We expressed results as odds ratios (ORs) with 95% confidence intervals (CIs), and we considered *P-*values < 0.05 as statistically significant.

## RESULTS

The study included records from 2,909 women diagnosed with GDM between 2021 and 2023. Women were categorized into four groups based on their PPBMI: underweight (*n*=339; 11.65%), normal-weight (*n*=2,166; 74.48%), overweight (*n*=357; 12.28%), and obese (*n*=47; 1.62%).

We found significant differences in maternal and neonatal outcomes across BMI categories (Table-I). Women aged 20-30 were predominantly underweight (54.57%), while women aged 30-40 had mostly normal weight (56.69%) or were overweight (64.71%), with the highest proportion of obese women in this age group (68.09%). In terms of the delivery mode, underweight women (73.45%) had higher rates of normal delivery, while cesarean sections were most common among obese women (72.34%). The incidence of having an SGA infant was higher in normal-weight (24.70%) and overweight (21.57%) women respectively. In terms of neonatal outcomes, the risk of macrosomia was most frequent in the obese group (10.64%), while LBW was more common in underweight women (7.37%). NICU admissions were highest among newborns of underweight (9.73%) and obese (6.38%) women. Maternal complications also showed significant variation. Pregnancy-induced hypertension (PIH) was most common in obese (23.40%) and overweight (12.32%) women, while underweight women had the lowest risk (1.18%).

**Table-I:** Neonatal and Maternal characteristics and their prevalences in pre-pregnancy BMI groups.

Variables	Pre-pregnancy BMI category				p-value

	Underweight (n = 339)	Normal weight (n = 2166)	Overweight (n = 357)	Obese (n = 47)	
***Demographic variables***					
Age (years)					
20–30	185 (54.57)	792 (36.57)	100 (28.01)	14 (29.79)	<0.01^*^
30–40	147 (43.36)	1228 (56.69)	231 (64.71)	32 (68.09)	
40–50	7 (2.06)	146 (6.74)	26 (7.28)	1 (2.13)
***Gestational weeks at delivery (weeks)***					
< 37	20 (5.90)	133 (6.14)	18 (5.04)	4 (8.51)	0.32
37-40	234 (69.03)	1493 (68.93)	270 (75.63)	34 (72.34)	
< 40	85 (25.07)	540 (24.93)	69 (19.33)	9 (19.15)
***Delivery Mode***					
Normal	249 (73.45)	1272 (58.73)	161 (45.10)	13 (27.66)	<0.01^*^
Caesarian section	90 (26.55)	894 (41.27)	196 (54.90)	34 (72.34)
***Neonatal complications***					
***Size at Gestational Age***					
SGA	15 (4.42)	535 (24.70)	77 (21.57)	1 (2.13)	<0.01^*^
Normal GA	261 (76.99)	1271 (58.68)	236 (66.11)	40 (85.11)	
LGA	63 (18.58)	360 (16.62)	44 (12.32)	6 (12.77)
***Neonatal weight***					
LBW	25 (7.37)	100 (4.62)	12 (3.36)	1 (2.13)	0.07
Normal birth weight	308 (90.86)	1934 (89.29)	310 (86.83)	41 (87.23)	
Macrosomia	6 (1.77)	132 (6.09)	35 (9.80)	5 (10.64)
***Neonatal Jaundice***					
Yes	106 (31.27)	653 (30.15)	104 (29.13)	19 (40.43)	<0.01^*^
No	233 (68.73)	1513 (69.85)	253 (70.87)	28 (59.57)
***NICU admission***					
Yes	33 (9.73)	1 (0.05)	4 (1.12)	3 (6.38)	<0.01^*^
No	306 (90.27)	2165 (99.95)	353 (98.88)	44 (93.62)
***Neonatal asphyxia***					
Low	336 (99.12)	2096 (96.77)	346 (96.92)	47 (100)	<0.01^*^
Moderate	3 (0.88)	57 (2.63)	10 (2.80)	0 (0)	
Severe	0 (0)	13 (0.60)	1 (0.28)	0 (0)
***Maternal complications***					
PIH					
Yes	4 (1.18)	112 (5.17)	44 (12.32)	11 (23.40)	<0.01^*^
No	335 (98.82)	2054 (94.83)	313 (87.68)	36 (76.60)
***PROM***					
Yes	100 (29.50)	607 (28.02)	94 (26.33)	6 (12.77)	0.37
No	239 (70.50)	1559 (71.98)	263 (73.67)	41 (87.23)
***Hyperthyroidism***					
Yes	8 (2.36)	58 (2.68)	4 (1.12)	0 (0)	0.12
No	331 (97.64)	2108 (97.32)	353 (98.88)	47 (100)
***Hypothyroidism***					
Yes	19 (5.60)	96 (4.43)	15 (4.20)	6 (12.77)	0.08
No	320 (94.40)	2070 (95.57)	342 (95.80)	41 (87.23)
***Placental abruption***					
Yes	6 (1.77)	27 (1.25)	2 (0.56)	2 (4.26)	0.13
No	333 (98.23)	2139 (98.75)	355 (99.44)	45 (95.74)
***Puerperal infection***					
Yes	0 (0)	2 (0.09)	0 (0)	0 (0)	0.82
No	339 (100)	2164 (99.91)	357 (100)	47 (100)
***Chorioamnionitis***					
Yes	10 (2.95)	83 (3.83)	16 (4.48)	3 (6.38)	0.29
No	329 (97.05)	2083 (96.17)	341 (99.52)	44 (93.62)	

Multivariate regression analysis, adjusted for maternal age, gravidity, parity, neonatal gender, and pre-term history, showed that underweight women presented a significantly lower risk of SGA neonates and PIH, with the adjusted ORs of 0.11 (0.07 – 0.18) and 0.26 (0.09 – 0.72), respectively, and significantly higher risk of LBW newborns [adjusted OR of 3.40 (1.83 – 5.49)]. Conversely, overweight and obese women had a higher risk of PIH and macrosomia. Underweight PPBMI status was associated with a significantly lower risk of neonatal asphyxia, with an adjusted OR of 0.12 (0.03-0.41). (Table-II)

**Table-II:** Associations between pre-pregnancy BMI categories and outcomes.

Outcomes	Underweight	Normal weight	Overweight/Obese
Caesarian section n (%)	90 (26.55)	894 (41.27)	230 (56.93)
Unadjusted OR (95%CI)	0.51 (0.39 – 0.66)*	Ref	1.88 (1.51–2.33)*
adjusted OR (95%CI)	0.55 (0.41 – 0.72)*	Ref	1.62 (1.28 – 2.04)*
PIH n (%)	4 (1.18)	112 (5.17)	55 (13.61)
Unadjusted OR (95%CI)	0.21 (0.08 – 0.59)*	Ref	2.89 (2.05 – 4.06)*
adjusted OR (95%CI)	0.26 (0.09 – 0.72)*	Ref	2.96 (2.07 – 4.24)*
PROM n (%)	100 (29.50)	607 (28.02)	100 (24.75)
Unadjusted OR (95%CI)	1.07 (0.83 – 1.38)	Ref	0.84 (0.66 – 1.08)
adjusted OR (95%CI)	1.01 (0.77 – 1.33)	Ref	0.99 (0.77 – 1.29)
Hyperthyroidism n (%)	8 (2.36)	58 (2.68)	4 (0.99)
Unadjusted OR (95%CI)	0.87 (0.41 – 1.58)	Ref	0.36 (0.13 – 1.00)
adjusted OR (95%CI)	0.87 (0.39 – 1.65)	Ref	0.36 (0.13 – 1.04)
Hypothyroidism n (%)	19 (5.60)	96 (4.43)	21 (5.19)
Unadjusted OR (95%CI)	1.28 (0.77 – 2.12)	Ref	1.18 (0.72 – 1.91)
adjusted OR (95%CI)	1.39 (0.81 – 2.36)	Ref	1.14 (0.69 – 1.87)
Placental abruption n (%)	6 (1.77)	27 (1.25)	4 (0.99)
Unadjusted OR (95%CI)	1.42 (0.58 – 3.48)	Ref	0.79 (0.27 – 2.27)
adjusted OR (95%CI)	1.34 (0.50 – 3.54)	Ref	0.80 (0.27 – 2.34)
Preterm delivery n (%)	20 (5.90)	133 (6.14)	22 (5.44)
Unadjusted OR (95%CI)	1.01 (0.81 – 1.26)	Ref	0.83 (0.67 – 1.02)
adjusted OR (95%CI)	1.07 (0.79 – 1.45)	Ref	0.86 (0.64 – 1.14)
LGA n (%)	63 (18.58)	360 (16.62)	50 (12.37)
Unadjusted OR (95%CI)	1.14 (0.85 – 1.54)	Ref	0.71 (0.51 – 0.97)*
adjusted OR (95%CI)	1.03 (0.71 – 1.51)	Ref	0.81 (0.55 – 1.20)
SGA n (%)	15 (4.42)	535 (24.70)	78 (19.30)
Unadjusted OR (95%CI)	0.13 (0.08 – 0.22)*	Ref	0.62 (0.48 – 0.81)*
adjusted OR (95%CI)	0.11 (0.07 – 0.18)*	Ref	0.67 (0.51 – 0.86)*
Macrosomia n (%)	6 (1.77)	132 (6.09)	40 (9.90)
Unadjusted OR (95%CI)	0.27 (0.12 – 0.63)*	Ref	1.69 (1.17 – 2.45)*
adjusted OR (95%CI)	0.33 (0.14 – 0.77)*	Ref	1.43 (0.98 – 2.10)
Low birth weight n (%)	25 (7.37)	100 (4.62)	13 (3.21)
Unadjusted OR (95%CI)	1.64 (1.05 – 2.59)*	Ref	0.68 (0.38 – 1.23)
adjusted OR, (95%CI)	3.40 (1.83 – 5.49)*	Ref	0.46 (0.23 – 0.94)*
Neonatal Asphyxia n (%)	3 (0.88)	70 (3.23)	11 (2.72)
Unadjusted OR (95%CI)	0.28 (0.09 – 0.85)*	Ref	0.81 (0.47 – 1.40)
adjusted OR, (95%CI)	0.12 (0.03 – 0.41)*	Ref	0.83 (0.45 – 1.50)

Pre-pregnancy BMI was adjusted for maternal age, gravidity, parity, neonatal gender, and pre-term history.

* p <0.05

## DISCUSSION

This study demonstrated a significant impact of pre-pregnancy body mass index on the pregnancy and neonatal outcomes in women with GDM. Our results showed that in GDM pregnancies, underweight PPBMI is associated with considerably lower risks of PIH and cesarean delivery but higher risks of LBW in offspring. In contrast, overweight and obese women are at higher risk of PIH and elevated incidence of cesarean sections and macrosomia.

PPBMI is a significant predictor of various maternal and neonatal outcomes.^14^-^16^ Previous studies have showed that in women with GDM, maintaining a normal BMI before pregnancy can mitigate the risks associated with both low and high BMI.^17^-^20^ The potential confounders in this study included maternal age, gravidity, parity, neonatal gender, and preterm history. These confounders can influence pregnancy outcomes independently of BMI and GDM. For instance, advanced maternal age is associated with increased risks of gestational complications,^18^ whereas high parity can influence maternal health and pregnancy outcomes.^19^ To isolate the effects of BMI on pregnancy outcomes, we adjusted for these confounders using multivariable logistic regression models.

Our observation of the impact of PPBMI on pregnancy and neonatal outcomes in the study cohort agrees with the results of a previous study^10^ conducted on women without GDM in Hunan Province, China. In both cases, deviations from the normal BMI range were associated with increased risks of adverse pregnancy outcomes. While the previous study^10^ identified general risks associated with abnormal BMI values, our findings further underscore the heightened risks of adverse outcomes in women with GDM. This distinction is critical as it indicates the need for more intensive monitoring and tailored interventions for this high-risk group. The presence of GDM introduces additional complexities to pregnancy, making it essential to address both BMI and glucose control to improve pregnancy outcomes.^20^

In our study, excessive PPBMI was linked to a higher incidence of macrosomia in offspring, which is in agreement with the previous research.^21^ A study by Yogev and Langer^22^ demonstrated that newborns of obese women had an over two-fold higher risk of macrosomia than those of women with normal weight. Such an association between high PPBMI and adverse outcomes may be explained by several mechanisms. Previous studies showed that elevated BMI values are associated with increased insulin resistance and inflammation, exacerbating the metabolic challenges in women with GDM.^23^,^24^ Additionally, in GDM, excessive amounts of glucose that are passed through the placenta into the fetal circulation are stored as body fat, leading to macrosomia.^25^

Conversely, low BMI values in our study were associated with a higher risk of LBW neonate. This observation may reflect inadequate nutritional reserves, affecting fetal growth and increasing the risk of LBW and preterm deliveries.^26^,^27^ Additionally, rigorous glucose monitoring in GDM patients, regardless of their initial PPBMI, may lead to a nutritional imbalance in underweight patients that may result in fetal growth restriction.^28^ A retrospective cohort study by Kim et al.^29^ reviewed data from 946 women with GDM and showed that underweight women with GDM were at a higher risk of having SGA neonates. These results differ from our observation.

This study showed that while the risk of having an LBW offspring was higher in the underweight PPBMI category of women, no such association was found between the underweight status and the risk of neonatal SGA. This discrepancy may be due to the difference in the sample sizes between the studies and variability in the differential diagnosis of LBW and SGA. Future studies should assess the risk of both LBW and SGA in underweight women with GDM. Our results highlight the importance of achieving a balanced BMI before attempting to become pregnant to support optimal maternal and fetal health and further emphasize the need for tailored monitoring of this particular group of women with GDM.

We showed that overweight and obese PPBMI status was associated with an increased incidence of PIH, which is consistent with previous research. GDM is a known risk factor for gestational hypertension.^30^ A study by Lewandowska et al.^31^ reported that higher values of PPBMI were greater risk factors for PIH (adjusted OR = 4.94; *p* < 0.001) and preeclampsia (adjusted OR = 8.61; *p* < 0.001) than excessive gestational weight gain. The mechanistic connection between GDM and PIH is still not clear. Research suggests that abnormally increased glucose concentrations during GDM pregnancy may seriously impact lipid profiles. In turn, excessive lipid accumulation inside endothelial cells may lead to endothelial dysfunction and hypertension.^30^ The strengths of this study include its large sample size from a group of patients in the pre-pregnancy health check program, a detailed BMI categorization, and robust statistical adjustments for confounders. Focusing on a specific high-risk group (women with GDM) also provides targeted insights.

### Limitation:

The study’s retrospective nature may have introduced recall bias and limited our ability to establish causality. Although we collected the data prospectively, the accuracy and completeness of the data cannot be guaranteed due to the retrospective nature of the analysis. Another limitation is the reliance on self-reported data for variables such as gravidity, parity, and pre-term history, which can be inaccurate due to recall bias or intentional misreporting.

This could have affected the categorization of BMI values and the study’s findings related to BMI-related risks. In addition, we failed to include detailed data on glycemic control during pregnancy, which is a critical factor in managing GDM and influencing pregnancy outcomes.^30^ Lastly, the number of obese women in our study was relatively small compared to the number of women in the other BMI categories, this may have limited the statistical power of our analysis to detect differences and draw definitive conclusions about the risks associated with obesity in women with GDM.

Future research should address these limitations by incorporating prospective study designs, including diverse populations, and collecting comprehensive data on glycemic control and long-term outcomes. Expanding the study to multiple regions and increasing the sample size for all BMI categories, especially the obese group, would enhance the generalizability and robustness of the findings.

## CONCLUSION

The study revealed that pre-pregnancy BMI significantly impacts pregnancy outcomes in women with GDM, with both low and high BMI linked to higher risks of adverse maternal and neonatal outcomes. Our results highlight the need for pre-pregnancy counseling and interventions to achieve a healthy BMI, particularly for women with GDM, to reduce compounded risks and enhance maternal and neonatal health.

### Authors’ contributions:

**LZ:** Was involved in the design of the study, writing of the manuscript

**QL**, **JL**, **JS**, **ZC** and **LL:** Contributed to the data collection, data analysis and interpretation.

**LL:** Contributed to literature search, the critical revision and validation.

All authors are responsible for the integrity of the study and have read and approved the final manuscript.
